# Supplementary material for: Alternate Ultrasound/Microwave Digestion for Deep Eutectic Hydro-distillation Extraction of Essential Oil and Polysaccharide from Schisandra chinensis (Turcz.) Baill
Source: Molecules. 2019 Apr 2;24(7):1288. doi: 10.3390/molecules24071288 (PMC6479861; doi:10.3390/molecules24071288)
Supplement: Supplementary file 1 [file molecules-24-01288-s001.pdf]

molecules

Article

Alternate Ultrasound/Microwave Digestion for Deep Eutectic Hydro-distillation Extraction of Essential Oil and Polysaccharide from *Schisandra chinensis* (Turcz.) Baill

Jun-Han Li, Wei Li, Sha Luo, Chun-Hui Ma * and Shou-Xin Liu *

College of Material Science and Engineering, Northeast Forestry University, 150040, Harbin, China; nefulijunhan@163.com (J.-H.L.); liwei19820927@126.com (W.L.); luo.sha.85@163.com (S.L.);

* Correspondence: mchmchmchmch@163.com (C.-H.M.); liushouxin@126.com (S.-X.L.); Tel.: +86-451-82191204(C.-H.M.); Tel.: +86-451-82191502 (S.-X.L.)

Supplementary Information

Table S1. The credibility analysis of the regression equations for ultrasound-assisted extraction.

Index mark	Extraction yield of polysaccharides
Std. Dev.	0.86
Mean	7.75
C.V. %	11.09
PRESS	19.30
R-Squared	0.7853
Adjust R-Squared	0.7450
Predicted R-Squared	0.6489
Adequacy Precision	14.606

Table S2. The credibility analysis of the regression equations for microwave-assisted extraction.

Index mark [a]	Extraction yield of polysaccharides
Std. Dev.	0.47
Mean	7.69
C.V. %	6.16
PRESS	16.96
R-Squared	0.9397
Adjust R-Squared	0.8854
Predicted R-Squared	0.5435
Adequacy Precision	14.858
